# Memoirs of the Medical Society of Observation, of Paris

**Published:** 1838-01

**Authors:** 


### Art. X.

*Mémoires de la Société Médicale d'Observation, de Paris.* Tome premier. —*Paris,* 1836. 8vo. pp. 495.

*Memoirs of the Medical Society of Observation, of Paris.* Volume Ist. —*Paris,* 1836. 8vo. pp. 495.

This volume is the first proceeding from a small Society, founded by a few medical students in 1832, for the professed purpose of improving the science of observation in medicine, which they considered as extremely neglected and underrated; believing it, as they say, to be "contre l'opinion commune," difficult, and requiring a long apprenticeship; although it might perhaps be objected to these ardent and able reformers that the common opinion is not quite so erroneous as they represent it. Out of the forty or fifty names which compose the new Society, a few only are known and distinguished. M. Louis is the perpetual president, and MM. Chomel and Andral are the *honorary* presidents: among the rest, we observe some of considerable celebrity; but, as must happen in all societies, a few whose names carry little recommendation with them.

M. Louis is, of course, put into the front rank: he writes the preface, introduces his friends, French, English, American, and Genevese, the "young medicine" of the existing world; supplies an article of sixty pages on the Examination of the Sick and the Investigation of General Facts; and another of 100 pages on Emphysema of the Lungs. M. Maunoir gives a contribution of 100 pages on Cataract; and M. Bizot inserts 150 pages on the Heart and Arterial System in Man, divided, after the manner of the old theologians, into three parts, each part into chapters, and each chapter into articles; whilst to the whole are prefixed an "avertissement" and a preface. The remaining pages, about 100, are filled up by M. Marc d'Espine, with an analytic memoir, "sur l'Orchite Blennorrhagique."

Notwithstanding some degree of arrogance of tone, however, the plan of this Society is admirable. Its main object being to form observers, cases are contributed by the members at the weekly meetings, which are read and criticised; the omissions are commented upon, and the means of investigation into facts difficult to be established are freely enquired into. Thus, all the members are, it is said, gradually so improved as medical reporters, that the facts they relate acquire all the certainty which is necessary, and become part of groups to be hereafter analyzed by the Society. As to the method to be pursued in order to pass from particular to general facts, the necessity of the numerical analysis has always appeared to the members to be so evident, that no discussion, says M. Louis, has ever taken place on the point. It is, we confess, with respect to this latter particular that we have a slight distrust of some of the members of the Society of Observation. To maintain the character to which they aspire, it is at all events necessary that they should carefully distinguish the statistical and practical boundaries of the numerical method. Observers of one set of medical facts may easily group them, and with some statistical utility; but the observation necessary for the safe treatment of diseases comprehends many circumstances which distinguish every individual case from the rest. This view has been ably set forth by M. Double; and, although we doubt not that his notions are thought of small account by the professed observers and numerators, we believe the voice of the veteran is that of sage experience.

The perpetual president of the Society proposes, every year, one or more subjects of study, in which all the members are invited to take a part: a subject being fixed upon, some member presents a plan for its observation, and this plan is discussed and determined by the members. M. Bizot's treatise on the pathological alterations of the arterial system and of the heart was commenced according to this arrangement; and is certainly a striking proof of its value.

M. Louis' essay in this volume, on the Examination of the Sick and on the Investigation of General Facts, is intended as an exposition of the principles maintained by the Society. It very appropriately begins with a general, and by no means a just, condemnation of the physicians of antiquity and also of modern times, as bad observers and imperfect describers. Having advanced this indiscriminate remark with an air of remarkable satisfaction, M. Louis proceeds to account for it; and the reason of the lamentable deficiency is, of course, no other than the want of the numerical method. M. Louis thinks medicine may be advanced and rendered certain, just as chemistry may be and has been, by reckoning *every thing*. To take every thing into account is doubtless important to medical observation; but between chemical facts and medical facts there is surely small analogy. Atoms, and grains, and cubic inches are always the same, which cannot be rigorously said of any single symptom. A cough may not be weighed, nor the exact extent of a pain meted out, nor the combination of symptoms in a fever determined by the atomic theory. It is but fair to M. Louis to state, that he endeavours to justify his sweeping censure of all past observers by mentioning that Pinel intrusted to very young men the task of collecting the facts on which he founded his theories; and that, fifteen years ago, when he (M. Louis) persevered in the examination and collection of facts, being no longer a mere medical student, his application excited, on that account, so much surprise and pity, that it required some courage to go on with it. Greatly, indeed, was it to the honour of M. Louis that he did go on with it. Whatever he may say and think in reference to the numerical method, he has not himself forgotten its most useful applications, but has rendered them really available to the advancement of sound medical knowledge.

M. Louis points out the importance of a previous acquaintance with the age, occupation, usual diet, habitual constitution, mode of life, ordinary state of body, and former illnesses of the patient whose case is to be investigated; and he warns the examiner against the many sources of deception to be avoided. The object of our examination, he justly says, is not solely to verify the descriptions of authors, but to acquire knowledge; and each malady should be studied, in particular instances, under the greatest number of points of view possible. He dwells strongly on the two points of determining when the disease commenced, and what was the actual state of the patient's health previous to the attack; giving as instances of the important conclusions to be drawn from these circumstances, that of pleurisy, and that of erysipelas of the face, occurring in persons previously in good or in disordered health, by which the prognosis would be considerably modified. Each symptom is then, says M. Louis, to be studied in the order of its development. Every function is separately to be investigated.

"Besides this, it is not sufficient, in the actual state of the science, to have ascertained the beginning, the degree, and the progress of the symptoms: their study, to be complete, requires a further precision, the importance of which is easily understood, and need only be briefly remarked upon. Suppose, for example, that the abdomen is, or has been, the seat of pain, it is not sufficient to make known its existence, character, and commencement; we must ascertain, if possible, the point of its commencement, since this may be the exact point from which the disease commenced; as may be seen in the typhoid affection, in which the pain of the abdomen ordinarily begins in the iliac fossa on the right side, or in the point corresponding to the first patches of the glands of Peyer which have undergone change. I may say the same, and for the same reasons, of pains of the chest, of percussion, and of thoracic auscultation. It would be quite insufficient, for example, to note that the thorax returns an obscure sound on the right side, anteriorly or posteriorly: the point where the dull sound is rendered must be precisely indicated, at the summit, at the base, or the sides, and its extent and degree should be marked. The same remarks apply to the different kinds of râle, and particularly to the subcrepitous. Merely to ascertain its presence, for instance, in acute pulmonary catarrh, in which it is so frequently observed, would be almost to do nothing, or to pass over one of the most important points in the history of this kind of râle. Observation, in fact, teaches that, at its commencement, it only exists in the posterior and lower parts of the chest; by which a very essential difference is established between catarrh and phthisis. By daily studying the subcrepitous râle, and noting exactly the part of the thorax where it ceases, it is soon perceived that it extends itself from the base to the summit, without interruption; and we thus establish, by the only means of observation which we yet possess, that pulmonary catarrh extends like other affections of the same kind." (P. 11.)

The same minute attention, M. Louis maintains, is necessary in the prosecution of pathological anatomy. Every organ should be examined, and its condition, healthy or diseased, carefully observed; the object of the examinations being not merely to verify the seat of the disease, its nature, and complications, but to attain a fuller knowledge of the laws of the animal economy, which can in no other way be attained than by an examination of every organ, whatever may have been the cause of death. We owe, he observes, to pathological anatomy, cultivated after this method, the knowledge of the fact that, in a subject above fifteen years of age, we never find tubercles or grey semi-transparent granulations in any organ, without the same being existent, and often far advanced, in the lungs. In the same manner it has been established that ulcerations of the pharynx, of the œsophagus, and of the small intestines, belong exclusively (with the exception of syphilis,) to two affections, severe fevers and phthisis; and that ulcerations of the larynx and trachea, particularly the latter, only occur (with the same exception,) in phthisical subjects. The knowledge of such laws may occasionally serve to interpret such diseases, in the absence of ordinary symptoms.

"Thus, that form of peritonitis which is chronic in its character from the commencement, is, according to all the facts I have been able to collect, constantly tubercular in adults, or in patients from fifteen years old to old age, or associated with grey semi-transparent granulations, developed upon or under the peritoneum. But, as I have just observed, neither of these lesions exists in any organ without being also observed in the lungs; so that a chronic peritonitis being actually established, we may recognize phthisis, independently of symptoms connected with the state of respiration, and even in their absence; meaning the existence of more or fewer tubercles, or grey semi-transparent granulations, in the lungs. If there are any exceptions to this law, they are very rare. I have more than once announced the existence of phthisis in patients who had all the symptoms of chronic peritonitis, without any alteration of the pulmonary parenchyma appreciable by the stethoscope or by percussion; and even in patients who had no cough: a diagnosis which was sometimes deemed very uncertain, or even rash, but proved to be correct, and which I could not have renounced without abandoning the laws of the animal economy and science itself; for the laws constitute the science." (P. 17.)

In the rules which M. Louis lays down for the investigation of the predisposing and exciting causes of disease, in each patient submitted to examination, we find the same exactness of method, and the same contempt of labour for the great object of increasing the knowledge of all that appertains to disease. He is careful, throughout, to point out every proper object of enquiry, and he notices every probable source of deception.

The second chapter of M. Louis's paper (for it seems the habit of all the members of the Society to divide their essays into chapters,) commences with an earnest repetition of the advice, already many times given in the first, to observe *all* the symptoms in each case, and not those which are merely diagnostic or proper to the malady supposed to be present. He lays it down as a principle that truth is not to be divined, but to be found, and that nature is a problem only to be solved by means of a sufficient number of exact facts. These facts being numerous, are to be arranged in proper groups, according to their similitude, and the identity of the affection they indicate or disprove. It is of little consequence with what symptom the enquiry begins; the first thing being to ascertain in what proportion of cases it shews itself, without which physicians are not justified in speaking of it as rare or as frequent; expressions implying that they have counted, when they have not. Mere counting, however, he admits, may lead to absurd conclusions; each symptom enumerated must be considered in relation to the age, sex, and various circumstances of the patient; but he still maintains that it is necessary to count; and he denies the possibility of avoiding error, or arriving at exact conclusions, by any other method.

Now, although we do not dispute the general correctness of these remarks, it is impossible not to see that not only in medicine, but in the general conduct of life, there is much wisdom acquired, and of a practical kind, on which men are obliged to depend, and which was never acquired by this formal and mechanical process. What is called knowledge of character and experience of mankind is not learned by counting, but by reasoning from impressive events: it is a knowledge acquired by induction, not by numeration; by induction of like particulars, not by counting circumstances, including such particulars with various other particulars. A man who is addressed by a swindler, and who suspects, from certain characters of the address made to him, that the person making it is not an honest man, does not derive his suspicion from his tablets; he does not look at his memorandum-book to see, having been so addressed twenty times before, whether he was cheated eight times, or twelve times, or eighteen times, and whether in all the cases the swindler had the same manner, the same cast of countenance, used the same words, or more than half of the same words, or was of the same age, stature, embonpoint, countenance, and colour of hair. He attains the useful conclusion much more quickly, and with no less certainty, by inductions from looks and manner associated in his mind with villany: and we are convinced that, if we regard medicine in relation to the practical art for which it is chiefly studied, the laborious counter of facts would often be found too minute to be useful, and his processes both elaborate and deceptious. The patient would die before he had finished his observation, or, at least, before he had come to the end of his arithmetic. The most accurate and extensive tables of life, whatever number of years they may promise to fifty per cent. of men of a given age, cannot secure any one of the fifty from an accident at any moment of any one day; but each case in medical practice is as this one case of the fifty. Each case in the fifty is liable to contingencies affecting its character, and calling for a corresponding modification of the means employed. Therefore, although, for statistical purposes, it is evident that recourse may be had to the numerical method in all its formality and exactness, the mental enumeration followed in practice will ever be a shorter process, and perhaps must be shorter that it may be promptly serviceable. It may be objected that the short method of numeration is never rigidly correct, or is open to error; but it would require more faith in minute observers, and in tables, and all the long array of figures, than we possess, to impress us with a conviction that the longer process would prove infallible, even if adopted by innumerable observers, and continued for centuries. The almost universal distrust of other men's observations which now makes every man rely much upon his own experience, would not yield, in particular instances, to all the figures in the world. The practitioner would bleed or not bleed in pneumonia or in fever, not because so many millions had been successfully bled at the same age, period of disorder, &c., but because, by certain mental processes, no less valuable than tables of cast-up figures, he was convinced that bleeding would save or endanger life. To suppose the contrary, would make little more necessary to the practitioner than a moderate share of the perceptive faculties, and a book of tables, and every case would become a question in addition and subtraction. Superiority of understanding, the ready exercise of judgment, and good sense, would go for nothing. They would wholly disregard the philosophical truth advanced by M. Broussais, in his spirited letter to M. Risueno d'Amador, (*Rev. Méd., Juillet*, 1837, p. 141,) that the causes acting on the impressible animal economy are ever changing, in combinations or in kind, and would mechanically rule the future by the past. These objections are not merely theoretical. We have had sufficient opportunity of observing the observers, as they exclusively consider themselves, to know how the vast pile of details which they laboriously accumulate, shuts out from their view those general views which distinguish a good practitioner from a bad one; views which alone impart the quality of sagacity. The mistake of the numerators is, that they depreciate all calculation that is not reduced to figures, as if no one could reckon ten without counting his fingers. To use their own words, *more* and *less*, and *often* and *seldom*, have no meaning, unless we can say how many times *per cent.* They would object to the expression, *Many people suffered severely from the influenza last winter.* In the first place, what is *many?* Is it ten, twenty, thirty, or sixty per cent.? Secondly, what is *severely?* Thirdly, what is *influenza?* Fourthly, what season of the year can strictly be called *winter?* First prove all these things by tables. And yet we imagine many of our readers will think that the assertion may be safely relied upon, without all this apparatus; and that the laborious individual who sate down to solve all these problems before venturing to make the assertion, although he might become a "full man," would not by any means be a "ready" one. But readiness in practice is every thing. The most undeniable conclusions, if only carried in the pocket, are of no service to the sick. A man is seized with apoplexy, and the bystanders say his father died of the same malady. You give half an hour to the investigation of the apoplexy, and half an hour to investigating the age, habits of life, and mode of death of the father; and you become convinced that the disease before you is apoplexy. Should you bleed? You have some tables at home which show you how many people have been bled in apoplexy with good results; and these are connected with other tables which show you their ages, and these with others which shew in how many it was hereditary. The pupils are contracted, and the table shews only forty-nine per cent. of recoveries after bleeding when the the pupils were contracted; but the pulse is full and slow, and the tables shew fifty-five per cent. of recoveries where the pulse was slow and the patient bled:—then the breathing, the degree of intelligence;—more tables! Who is to decide amidst such thickly serried columns? At length you ascertain that fifty-nine per cent. of apoplexies at that time of life, and hereditary, are benefited by bleeding, and you resolve to bleed; but in the mean time the patient is dead. Still, the case will add another unit to the tables, and shew your accuracy of method. If the patient had been bled at once, he might have lived; but what would have become of science? and how could you have held up your head in the Medical Society of Observation?

If we take a case leaving more time for calculation, the advantage to the numerical methodists is not practically so great as they imagine. Suppose that we have been disciples of the school in which every fever is represented as a gastro-enteritis, this notion will of course very much affect our practice. To be delivered from this, observation is necessary, but numeration is of little importance. If we become convinced that fever exists in five cases, or in two, or in one in a hundred, without gastro-enteritis, the fact is as practically useful as if the proportion instead of five was fifty per cent. These are surely the facts which are to be weighed rather than counted. But, with all this, we admit, that for scientific purposes, and for the advancement of statistical knowledge, the numerical method is essential. Our objections apply only to its abuse; or to a reliance upon it in single cases, as the guide to safe practice.

The enumerators tell us, that fatty transformation of the liver is more frequent in phthisical women than in phthisical men; and ulceration of the epiglottis, larynx, and trachea more frequent in phthisical men than in phthisical women. This kind of fact will scarcely find general acceptance, resting wholly upon figures. It will always be considered doubtful. Now, if admitted, is its importance considerable? for no figures would convince any man of tolerable experience that the exceptions as regarded the ulcerations of the larynx, &c. were not so numerous as to make the arithmetical balance practically little worthy of regard. Yet M. Louis would call the person not keeping the account ever before him no observer, but, in derision, a "man of genius;" whilst the person who disregarded the laryngeal symptoms on arithmetical principles, and took his determined stand upon the liver, would be held in honour, as a man who knew that two and two made four.

"Up to this time," exclaims M. Louis, (for his language is rather declamatory in this essay), "people have never or rarely counted: well! of what malady, except some eruptive affections, in which it has been very imperfectly observed, of what malady do we know the mean duration? Books of pathology say, indeed, it is true, that the age, the temperament, &c. have an influence on the march and duration of maladies: but where is the rigorous proof of this assertion? Where is the measure of this influence? No where to be found. And how, in fact, could they be attained except in the mode which has been indicated." (P. 33.)

This kind of language reminds us of poor Dr. Armstrong. It is assuredly unjust to say that the mean duration of maladies in general is unknown. It is equally erroneous, if we may trust to our own reading, to say that books of pathology lay great stress on the age, or on the temperament, as influencing the *duration* of maladies. If M. Louis thinks there is no proof that the age and temperament influence the *progress* (or march) of maladies, it says little even for his boasted method of observation. Besides, there are many diseases of which if we know not even the mean duration, we should not attain to it by numeration. What numeration table would satisfy any experienced man concerning the probable duration of epilepsy, or of mania, or even of a continued fever, the duration of which so many circumstances may prolong. M. Louis does wisely to laugh at the "man of genius;" for his numerical method is found to be the idol and practical guide of the dull alone. But he does unwisely to vaunt himself so unseemly; for he throws his past and great merits somewhat into the shadow. He has gained a high place, but he has grown giddy.

It is with reluctance and concern that we make these comments upon the manner of M. Louis; and we regret it the more because we are convinced that his challenging way of bringing forward the numerical method will create in many readers an indisposition to acknowledge its unquestionable merits; for that many facts are taken upon trust, on small foundation, cannot be denied. The causes of diseases are often arbitrarily and often erroneously assigned; their symptoms are not always well distinguished; and, perhaps, above all, the foundations of therapeutics are extensively unsound, and require inspection, consideration, and even the correction of the numerical method. Exposed to this test, the general opinion of many medicines would, we believe, undergo considerable modification; but to make therapeutical experiments conclusive, we must have calm observers, not anxious to catch a little fleeting fame by magnifying the merits of a new compound; and, as another preliminary, of small dignity but vast importance, we must have authentic drugs. Unless our information is very incorrect, there are not many prescriptions faithfully prepared in the British dominions. We believe there is scarcely a medicine, however simple, which the chemist's art cannot imitate in cheap and base material. There are many industrious "commercial gentlemen" in the chemical line, whose section of the business it is to supply the materials of adulteration to the brewer, the baker, and the retailer of drugs; and yet we eat and drink with indifference, and physicians prescribe with calm satisfaction. We fear these evils will be proof even against the numerical method; for so long as factitious drugs are given, there are no credible therapeutics to be counted, and the medicinal treatment of diseases is overlaid with fallacies.

If ever the time should arrive when in the majority of instances patients receive from the druggist precisely what the physician prescribes, pure and unadulterated, real roots, pure powders, and unexceptionable extracts, the numerical conclusions must still be received with caution, and with a continual remembrance that two cases can seldom be in every respect alike. Nor will this caution really vitiate the just numerical conclusion, any more than the general conclusions to which every practitioner arrives as results of experience. The numerical conclusion ought even, if carefully attained, to be more exact in itself, although it may not be, and we believe will not prove to be, more exact or useful in reference to single cases, the event of which must ever be regulated by individual modifications and complications. "To group facts according to their resemblance, and then to count them," is unquestionably one of the most likely ways of advancing towards truth; but all depends upon the rigid correctness of the groups. The whole question is, whether he who registers his facts in tables will be better guided by the numerical majorities he finds on adding up his columns, than he who reflects and registers his reflections in his mind. If both registers were kept with equal correctness, there could be no question on this subject. The written register would be infallible. But in the mental register, we apprehend that the material resemblances alone would be registered; whilst in the more formal tables the groups would often comprehend discordant particulars, especially in relation to the constitution and habits of patients, and the practical conclusions would be unsafe. In estimating the average success of surgical operations (one of the applications of the numerical method mentioned by M. Louis to recommend it), we think the fallacy incidental to mere counting is obvious. We may seem to dwell too much on these points; but we regard nothing with so much apprehension as a supposed infallibility in any part of the art of medicine. It is the mechanical exactness of the numerical method, supposed by its supporters to be its strongest recommendation, that makes us suspicious of it.

M. Louis furnishes some illustrative tables, which the reader may consult with interest, if not with advantage. Their tendency is certainly not to remove what may be our prepossessions. Let any one consider the number of tables and sub-tables required to present accurate numerical results concerning twenty cases of continued fever alone. First, a table of symptoms, subdivided into a table of ages, habits of life, and duration of the malady; a table of tongues, with a sub-table of habitual states of tongues in a certain portion of the patients; a table of the continuance of the malady, with sub-tables of the accidental causes of prolongation; a table of pulses, with a sub-table of peculiarities;—these and half a dozen more tables and sub-tables of treatment, diet, and examinations after death, would alone be likely to lead to any accurate calculations. Whether they would really lead to much or not is a question with which it is unnecessary to harass ourselves, for we may rest assured that such a method of mental progression will never be tried, to adopt the numerical language, by ten per cent. of medical practitioners.

It was our intention to have added to this notice of M. Louis's preliminary essay, the analysis of some of the medical memoirs which follow it, several of which, being on subjects rigidly admitting the application of the numerical method, are excellent illustrations of its real value. Our limits, however, oblige us to postpone this to another number.

---

